# Triglyceride-glucose index predicts ventricular aneurysm formation in acute ST-segment elevation myocardial infarction

**DOI:** 10.3389/fendo.2025.1423040

**Published:** 2025-05-08

**Authors:** Xiaobin Zeng, Yanyu Zhang, Xiaoshuang Xie, Jianjun Lan, Shiyang Li

**Affiliations:** ^1^ Division of Cardiology, Panzhihua Central Hospital, Panzhihua, China; ^2^ Clinical Laboratory, Panzhihua Central Hospital, Panzhihua, China; ^3^ Department of Geriatrics, Panzhihua Central Hospital, Panzhihua, China; ^4^ Department of Geriatrics, Panzhihua Central Hospital Affiliated to Dali University, Dali, China

**Keywords:** triglyceride-glucose index, acute ST-segment elevation myocardial infarction, primary percutaneous coronary intervention, left ventricular aneurysm, predictive

## Abstract

**Background:**

The triglyceride–glucose (TyG) index has been confirmed to be a predictor of cardiovascular diseases. The present study aimed to assess the predictive value of TyG index for left ventricular aneurysm (LVA) formation and prognosis in patients with acute ST-segment elevation myocardial infarction (STEMI) who underwent primary percutaneous coronary intervention (PCI).

**Methods:**

This prospective study included 991 patients with acute STEMI who underwent primary PCI. Multivariable logistic regression and receiver operating characteristic (ROC) curve analysis were used to assess the predictive value of TyG index for LVA formation. Prognosis analysis was performed with cox proportional hazard regression.

**Results:**

The prevalence of LVA was 14.4%. A higher TyG index was associated with a greater incidence of LVA (23.1% vs. 11.8%, P< 0.001). The TyG index was also higher in the LVA group than in the non-LVA group (9.4 ± 0.9 vs. 9.0 ± 0.8, P<0.001). Multivariable logistic regression analysis revealed that the TyG index was independently associated with the risk of LVA [odds ratio (OR)= 2.4, 95% confidence interval (CI)= 1.51-3.82, P< 0.001]. The predictive value of the TyG index remained significant even after cross-validation by dividing the study population into a training set (OR= 2.32, 95% CI= 1.24-4.35, P= 0.009) and validation set (OR= 3.19, 95% CI= 1.42-7.19, P= 0.005). Higher TyG index was correlated with increased risk of cardiac death (HR= 2.17, P= 0.04). The maximal length and width of LVA were significantly increased in patients with TyG index ≥ 9.68 compared with < 9.68 (P< 0.001). The discriminant power of TyG index for LVA was 0.742, which was superior to both triglyceride (C statistic= 0.666) and fasting blood glucose (C statistic= 0.613). The combination of TyG index, left ventricular ejection fraction, gensini score, and left anterior descending artery as the culprit vessel could significantly improve the predictive ability (C statistic= 0.908).

**Conclusions:**

A higher TyG index was an independent predictor for LVA formation and increased risk of cardiac death in patients with STEMI who underwent primary PCI.

## Introduction

ST-segment elevation myocardial infarction (STEMI) is the most dramatic manifestation of acute myocardial infarction (AMI) associated with increased short- and long-term cardiac death (1). Left ventricular aneurysm (LVA) is a common and severe complication of AMI, characterized by the outward expansion of the infarcted myocardium during both systole and diastole (2). Patients with AMI and LVA have a six-fold higher cardiac death rate compared to those without LVA, primarily due to the higher incidence of arrhythmias, thromboembolic phenomena, congestive heart failure, and cardiac rupture (3, 4). Given the high incidence rate (10%-38% of patients with AMI) (5) and poor prognosis associated with LVA, it is crucial to identify risk factors that contribute to its formation for effective prophylactic treatment.

The triglyceride-glucose (TyG) index, which is calculated using fasting triglyceride and blood glucose level data, has been identified as a reliable biomarker for evaluating insulin resistance (IR) (6). Additionally, numerous studies have indicated an association between TyG index and cardiovascular diseases. In a prospective cohort study involving 823 patients, a high TyG index was found to be correlated with an increased risk for cardiac death and rehospitalization in patients with heart failure with preserved ejection fraction (7). Furthermore, Chen. et al. reported that the TyG index was associated with recurrent revascularization in patients with type 2 diabetes mellitus after percutaneous coronary intervention (8). Moreover, the TyG index has been demonstrated to be an effective prognostic factor and for risk stratification in patients with acute coronary syndrome (ACS) (9–13). More importantly, the molecular mechanisms of TyG index as a marker for predicting cardiovascular diseases have also been studied, and included metabolic fexibility, endothelial dysfunction, coagulation disorders, and smooth muscle cell dysfunction (9). However, no study has investigated the association between TyG index and the risk of LVA formation. As such, the purpose of the present study was to assess the predictive value of the TyG index for the risk of LVA formation in patients with acute STEMI in a sample of the Chinese population.

## Methods

### Study design and participants

This study was approved by the Review Board of Panzhihua central hospital (Sichuan, Panzhihua, China) and adhered to the principles of the Declaration of Helsinki. Written informed consents were obtained from all participants. A total of 1201 consecutive patients with acute STEMI, who underwent primary PCI at the Cardiology Division of Panzhihua Central Hospital between March 2018 and June 2023, were recruited. Acute STEMI was diagnosed based on the fourth universal definition of myocardial infarction (14), which includes the following criteria: typical chest pain lasting > 30 minutes, with new ST-segment elevation at the J point in at least two contiguous leads of > 2 mm (0.2 mV) in males or > 1.5 mm (0.15 mV) in females on admission electrocardiogram, and an increase in cardiac enzyme levels > 99th percentile cut-off point for cardiac troponin I (cTnI). The exclusion criteria consisted of non-ischemic cardiomyopathy (including hypertrophic and dilated cardiomyopathy), congenital heart disease, renal or liver failure, active infection, malignant tumors or a life expectancy < 1 year, thrombolytic therapy before admission, or loss to follow-up. Ultimately, a total of 991 patients were included in the association analysis.

### PCI procedure and definitions

Before primary PCI, all patients were administered aspirin (300 mg loading dose followed by 100 mg daily), clopidogrel (of 600 mg loading dose followed by 75 mg daily), or ticagrelor (180 mg loading dose followed by 90 mg twice daily). In addition, a bolus of unfractionated heparin (UFH) was administered intravenously at a dose of 70 U/kg of body weight. The primary PCI procedure was performed using either the standard radial or femoral approach, in accordance with current guidelines. A stent was deployed in the culprit artery of all patients. During the PCI procedure, the operator had the discretion to decide whether to use balloon pre-dilatation or post-dilatation, type of stents (bare metal or drug-eluting), and the application of thrombus aspiration. The glycoprotein IIb/IIIa receptor inhibitor tirofiban was initiated during the PCI procedure, at the operator’s discretion, with a 10 μg/kg intracoronary bolus followed by a 0.15 μg/kg/min intravenous infusion. A technically successful stent implantation was defined as residual postprocedural stenosis < 10% in the culprit lesion. Two independent experts assessed and confirmed the results of coronary angiograms, as well as the extent and degree of stenosis in each major coronary artery vessel. On discharge, medical therapy was prescribed based on individual patient condition and guideline recommendations for secondary prevention.

### Clinical follow-up and data collection

In this study, we conducted follow-up every 3 months through outpatient interview or by telephone contact. The primary endpoint was defined as development of LVA. The secondary endpoint was defined as cardiac deaths. Trained physicians blinded to the study’s purpose were charged with collecting information regarding patient demographics and clinical characteristics, including age, sex, hypertension, diabetes, smoking status, and medication used at discharge from the electronic medical recording system. Venous blood samples for laboratory investigations were collected from all patients on admission to the emergency room before primary PCI, as well as 8–12 h after PCI. To determine the peak value of cardiac enzyme levels, blood samples for troponin I (TnI) and lactate dehydrogenase (LDH) were obtained from a peripheral vein, after admission to the intensive coronary care unit, every 12 h during the first 48 h and every 24 h during the remainder of the stay in the intensive coronary care unit. Fasting blood-glucose (FBG) and lipid levels were measured after PCI. The TyG index was calculated using the following equation (9): In (fasting triglyceride [mg/dl] × FBG [mg/dl])/2.

### Definitions

The diagnosis of left ventricular aneurysm (LVA) was performed in accordance with the protocol outlined in the Coronary Artery Surgery Study (CASS) (15). The criteria for diagnosing LVA included the following: (I) bulging of the left ventricular wall during both diastole and systole, exhibiting either akinesia or dyskinesia; (II) clear demarcation of the infarcted segment; and (III) absence of trabeculation in the affected segment. After admission, two-dimensional transthoracic echocardiography (TTE) was performed within 3 days, and at 1 and 6 months during the follow-up period. LVA was diagnosed using TTE at the 6-month follow-up. Additionally, hypertension was defined as treatment for hypertension before admission or a blood pressure > 140/90 mmHg. Diabetes mellitus was defined as a fasting plasma glucose level > 7.0 mmol/L, a postprandial blood glucose level > 11.1 mmol/L, a hemoglobin A1c level > 6.5%, or treatment for diabetes mellitus. Smoking history was defined as having smoked > 2 pack-years and/or having smoked within the past year.

The Gensini score was calculated using the method developed by Celebi et al. (16). In summary, a severity score was assigned to each coronary stenosis based on the degree of narrowing (in %), as follows: ≤ 25% (1 point); 26% to 50% (2 points); 51% to 75% (4 points); 76% to 90% (8 points); 91% to 99% (16 points); and total occlusion (32 points). Additionally, each coronary stenosis score was multiplied by a factor that considered the importance of the lesion’s position in the coronary circulation: 5 for the left main coronary artery; 2.5 for the proximal segment of the left anterior descending coronary artery (LAD); 2.5 for the proximal segment of the circumflex artery; 1.5 for the mid-segment of the LAD; 1.0 for the right coronary artery, the distal segment of LAD, the posterolateral artery, or the obtuse marginal artery; and 0.5 for the other segments. The sum of the scores for each coronary segment yielded the Gensini score.

### Statistical analysis

Statistical analyses were performed using SPSS 26 (IBM Corporation Armonk, NY, USA). Descriptive statistics were expressed as number (percent) for categorical variables and as median and interquartile range (IQR) or mean and standard deviation (SD) for continuous variables. The best cut-off value of TyG index to stratify patients into two groups was determined to be 8.98 according to the receiver operating characteristic (ROC) curve analysis. Differences among groups were assessed using appropriate statistical tests, including the chi-squared, independent-sample *t*, or Mann–Whitney U tests. Univariate logistic regression analysis was used to examine the association between different variables and the risk for LVA formation. Variables with P< 0.05 in the univariate analysis were included in the multivariate logistic regression analysis. Cox proportional hazard regression models were performed to calculate the hazard ratio (HR) and 95% confidence intervals (95% CIs) for the associations between the TyG index and the prognosis of patients. All comparisons were two-sided, and differences with P< 0.05 were considered to be statistically significant.

## Results

### Patient characteristics

**Figure 1:**
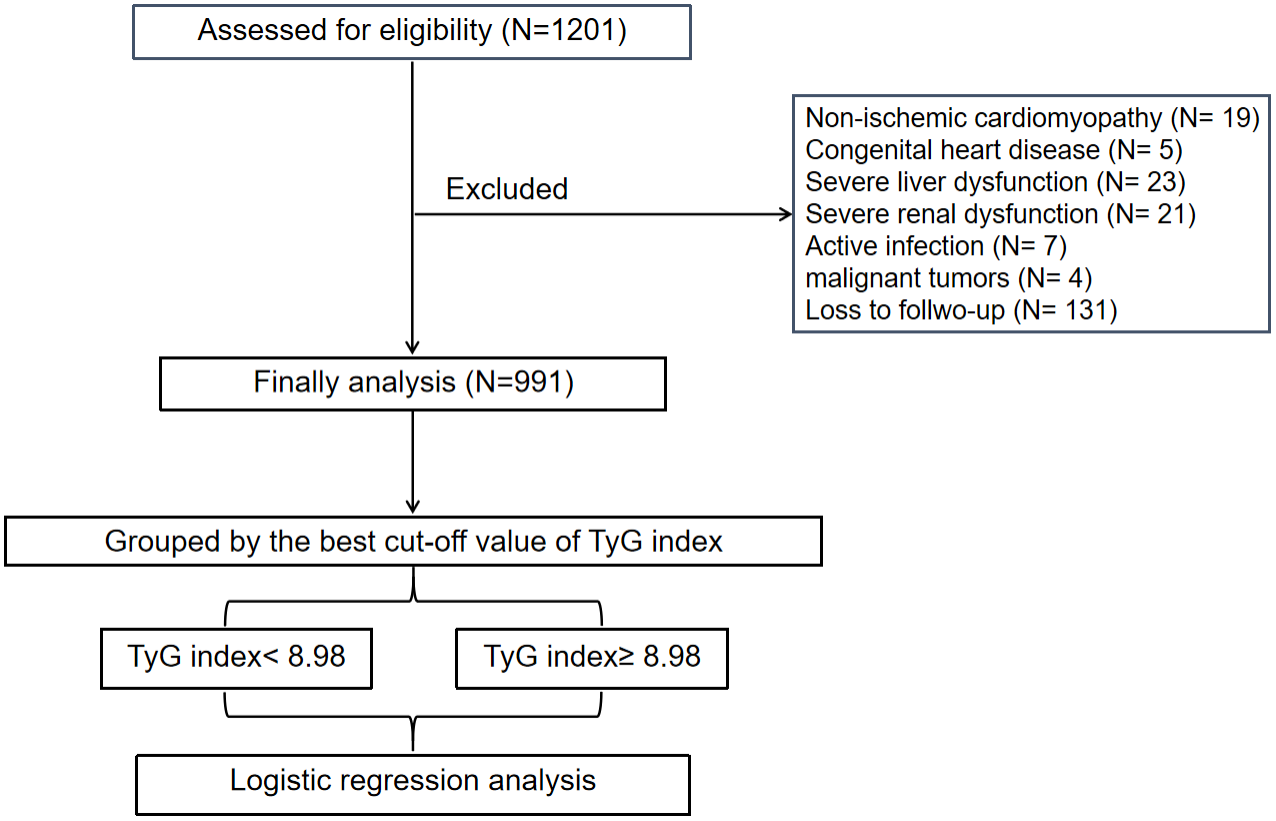
The overall workflow of methodology in the present study. TyG, triglyceride-glucose.

A flow-diagram depicting the overall workflow of the study is presented in **Figure 1**. Baseline characteristics of the cohort (n = 991) are summarized in **Table 1**. The mean (± SD) age of the study cohort (78.4% male) was 61.1 ± 12.8 years. Throughout the follow-up period for TTE, a total of 143 LVA incidents (14.4%) were observed.

**Table 1:** Baseline characteristics and laboratory findings of study population according to the presence of center ventricular aneurysmus.

Demographics	Total population (N= 991)	Non-LVA patients (N= 848)	LVA patients (N= 143)	P-value
Age, years	61.1 ± 12.8	60.2 ± 12.7	66.5 ± 11.9	<0.001
Male, n (%)	777 (78.4)	678 (80.0)	99 (69.2)	0.004
Hypertension, n (%)	553 (55.8)	464 (54.7)	89 (62.2)	0.094
Diabetes, n (%)	299 (30.2)	249 (29.4)	50 (35.0)	0.177
Smoking, n (%)	521 (52.6)	460 (54.2)	61 (42.7)	0.01
LVEF, %	56 (50–60)	57 (53–60)	43 (37–50)	<0.001
SBP, mmHg	129.3 ± 20.8	129.3 ± 21.0	128.9 ± 19.7	0.81
DBP, mmHg	80.8 ± 14.3	80.2 ± 14.6	83.9 ± 11.7	0.001
Heart rate, beats/min	83.3 ± 16.0	82.3 ± 15.6	89.1 ± 16.9	<0.001
Laboratory tests				
White blood cell count, 109/L	9.8 (7.7-12.0)	9.8 (7.7-11.9)	9.7 (7.5-12.4)	0.71
Red blood cell count, 109/L	4.5 ± 0.6	4.5 ± 0.6	4.4 ± 0.7	0.14
Neutrophil count, 109/L	7.6 (5.7-9.6)	7.4 (5.7-9.5)	7.9 (5.9-10.2)	0.262
Platelet count, 109/L	223.6 ± 68.5	224.7 ± 70.2	217.1 ± 57.1	0.22
Hemoglobin, g/L	138.2 ± 20.3	139.0 ± 20.1	133.8 ± 21.1	0.005
ALT, U/L	27.2 (17.6-48.4)	27.1 (17.8-48.2)	28.1 (17.2-51)	0.865
AST, U/L	55.7 (24.7-160)	55.6 (24.8-152)	62.7 (24.6-226.8)	0.202
HbA1c, %	6 (5.6-6.9)	6 (5.6-6.8)	6.1 (5.7-7.1)	0.083
Cr, umol/L	72.2 (62.1-87.2)	71.6 (62-87.1)	78.3 (66.5-90.3)	0.007
TC, mmol/L	4.7 ± 1.2	4.7 ± 1.2	4.9 ± 1.2	0.04
TG, mmol/L	1.4 (1.0-2.1)	1.5 (1.0-2.2)	1.3 (0.8-1.7)	<0.001
HDL-C, mmol/L	1.0 ± 0.3	1.0 ± 0.3	1.0 ± 0.3	0.63
LDL-C, mmol/L	3.1 ± 1.1	3.1 ± 1.1	3.4 ± 1.3	0.006
LDH, U/L	311 (202-556)	283 (199-533)	472 (235-712)	<0.001
Peak cTnI, ng/mL	19.3 (3.5-50)	15.1 (3.5-47.7)	26.8 (4.9-50)	<0.001
FBG, mg/dl	7.1 (5.9-9.6)	7.0 (5.8-9.3)	7.7 (6.4-12.1)	<0.001
TyG index	9.1 ± 0.8	9.1 ± 0.8	9.4 ± 0.8	<0.001
Medication at hospital discharge				
Aspirin	1583 (100.0)	1360 (100.0)	223 (100.0)	>0.999
Clopidogrel/Ticagrelor	1583 (100.0)	1360 (100.0)	223 (100.0)	>0.999
Statin, n (%)	984 (99.3)	844 (99.5)	140 (97.9)	0.108
β-blockers, n (%)	715 (72.1)	622 (73.3)	93 (65.0)	0.04
ACE inhibitors or ARB, n (%)	516 (52.1)	446 (52.6)	70 (49.0)	0.42
Aldosterone receptor blockers, n (%)	331 (33.4)	275 (32.4)	56 (39.2)	0.114
Thiazide or loop diuretic, n (%)	370 (37.3)	306 (36.1)	64 (44.8)	0.047
Coronary artery disease				
Gensini Score	71 (46-92)	66 (44-90)	88 (67-104)	<0.001
Culprit vessel-LAD, n (%)	492 (49.6)	380 (44.8)	112 (78.3)	<0.001

LVEF, center ventricular ejection fraction; SBP, Systolic pressure; DBP, Diastolic pressure; ALT, Alanine aminotransferase; AST, Aspartate aminotransferase; HbA1c, glycated hemoglobin; Cr, Creatinine; TC, total cholesterol; TG, triglyceride; HDL-C, high-density lipoprotein cholesterol; LDL-C, low-density lipoprotein cholesterol; LDH, Lactate dehydrogenase; FBG, fasting blood glucose; TyG, triglyceride-glucose; ACE, Angiotensin converting enzyme; ARB, angiotensin receptor blocker; LAD, center anterior descending artery.

Subsequently, baseline demographics and clinical characteristics of the patients in groups with and without LVA formation were compared. As shown in **Table 1**, patients in the LVA group were older, more likely to be female, and exhibited higher levels of diastolic blood pressure (DBP), heart rate, creatinine (Cr), total cholesterol (TC), low-density lipoprotein cholesterol (LDL-C), LDH, Peak cTnI, fasting blood glucose (FBG), TyG index, gensini score, and a higher proportion of thiazide or loop diuretic use, and LAD as the culprit vessel (P< 0.05) compared with those in the non-LVA group. Conversely, the proportion of patients who smoked and used β-blockers, the level of left ventricular ejection fraction (LVEF), hemoglobin, and triglycerides (TG) were lower in patients with LVA formation compared to those without LVA (P< 0.05).

According to the maximum Youden Index criterion and ROC curve analysis, the optimal cut-off value for the TyG index to stratify patients into two groups was determined to be < 8.98 and ≥ 8.98. As shown in **Table 2**, patients in the TyG≥ 8.98 group were younger, more likely to be female, and had higher systolic pressure (SBP), DBP, red blood cell count, hemoglobin, HbA1c, TC, TG, LDL-C, and FBG, as well as a higher prevalence of diabetes and LVA formation. In contrast, patients with TyG≥ 8.98 had lower levels of AST, high-density lipoprotein cholesterol (HDL-C), and Peak cTnI compared to those with TyG< 8.98.

**Table 2:** Comparison of the baseline characteristics and laboratory findings grouped by TyG index.

Demographics	TyG< 8.98 (N= 436)	TyG≥ 8.98 (N= 555)	P-value
Age, years	63.0 ± 11.7	59.6 ± 13.5	<0.001
Male, n (%)	355 (81.4)	422 (76.0)	0.041
Hypertension, n (%)	231 (53.0)	322 (58.0)	0.113
Diabetes, n (%)	62 (14.2)	237 (42.7)	<0.001
Smoking, n (%)	221 (50.7)	300 (54.1)	0.292
LVEF, %	56 (50-60)	56 (50-60)	0.669
SBP, mmHg	126.6 ± 18.5	131.4 ± 22.3	<0.001
DBP, mmHg	78.7 ± 12.9	82.4 ± 15.0	<0.001
Heart rate, beats/min	82.4 ± 17.0	84.0 ± 15.0	0.138
Laboratory tests			
White blood cell count, 109/L	9.6 (7.6-12.0)	9.9 (7.8-12.0)	0.397
Red blood cell count, 109/L	4.4 ± 0.6	4.6 ± 0.7	<0.001
Neutrophil count, 109/L	7.7 (5.5-10.0)	7.4 (5.8-9.2)	0.267
Platelet count, 109/L	219.1 ± 69.6	227.1 ± 67.5	0.068
Hemoglobin, g/L	135.4 ± 18.5	140.4 ± 21.5	<0.001
ALT, U/L	27.2 (15.8-46)	27.3 (18.8-49.9)	0.14
AST, U/L	68.5 (25.9-183.9)	50.4 (24.1-136.5)	<0.001
HbA1c, %	5.8 (5.5-6.2)	6.3 (5.8-7.9)	<0.001
Cr, umol/L	72 (61.8-88.8)	72.4 (62.2-86.4)	0.973
TC, mmol/L	4.3 ± 1.1	5.1 ± 1.3	<0.001
TG, mmol/L	0.9 (0.7-1.2)	2.0 (1.5-2.9)	<0.001
HDL-C, mmol/L	1.1 ± 0.3	1.0 ± 0.3	<0.001
LDL-C, mmol/L	2.9 ± 1.0	3.3 ± 1.2	<0.001
LDH, U/L	331 (207-592)	284 (197-532)	0.116
Peak cTnI, ng/mL	23.5 (4.9-50)	12.7 (2.5-50)	0.005
FBG, mmol/L	6.2 (5.5-7.3)	8.2 (6.7-11.6)	<0.001
TyG index	8.4 ± 0.5	9.6 ± 0.6	<0.001
LVA, %	41 (9.4)	102 (18.4)	<0.001
Medication at hospital discharge			
Aspirin	1262 (100.0)	321 (100.0)	>0.999
Clopidogrel/Ticagrelor	1262 (100.0)	321 (100.0)	>0.999
Statin, n (%)	434 (99.5)	550 (99.1)	0.658
β-blockers, n (%)	311 (71.3)	404 (72.8)	0.61
ACE inhibitors or ARB, n (%)	237 (54.4)	279 (50.3)	0.201
Aldosterone receptor blockers, n (%)	137 (31.4)	194 (35.0)	0.242
Thiazide or loop diuretic, n (%)	156 (35.8)	214 (38.6)	0.369
Coronary artery disease			
Gensini Score	69 (46-93)	71 (47-92)	0.859
Culprit vessel-LAD, n (%)	216 (49.5)	276 (49.7)	0.953

LVEF, center ventricular ejection fraction; SBP, Systolic pressure; DBP, Diastolic pressure; ALT, Alanine aminotransferase; AST, Aspartate aminotransferase; HbA1c, glycated hemoglobin; Cr, Creatinine; TC, total cholesterol; TG, triglyceride; HDL-C, high-density lipoprotein cholesterol; LDL-C, low-density lipoprotein cholesterol; LDH, Lactate dehydrogenase; FBG, fasting blood glucose; TyG, triglyceride-glucose; LVA, center ventricular aneurysm; ACE, Angiotensin converting enzyme; ARB, angiotensin receptor blocker; LAD, center anterior descending artery.

### TyG index and the incidence of LVA in patients who underwent primary PCI

**Figure 2:**
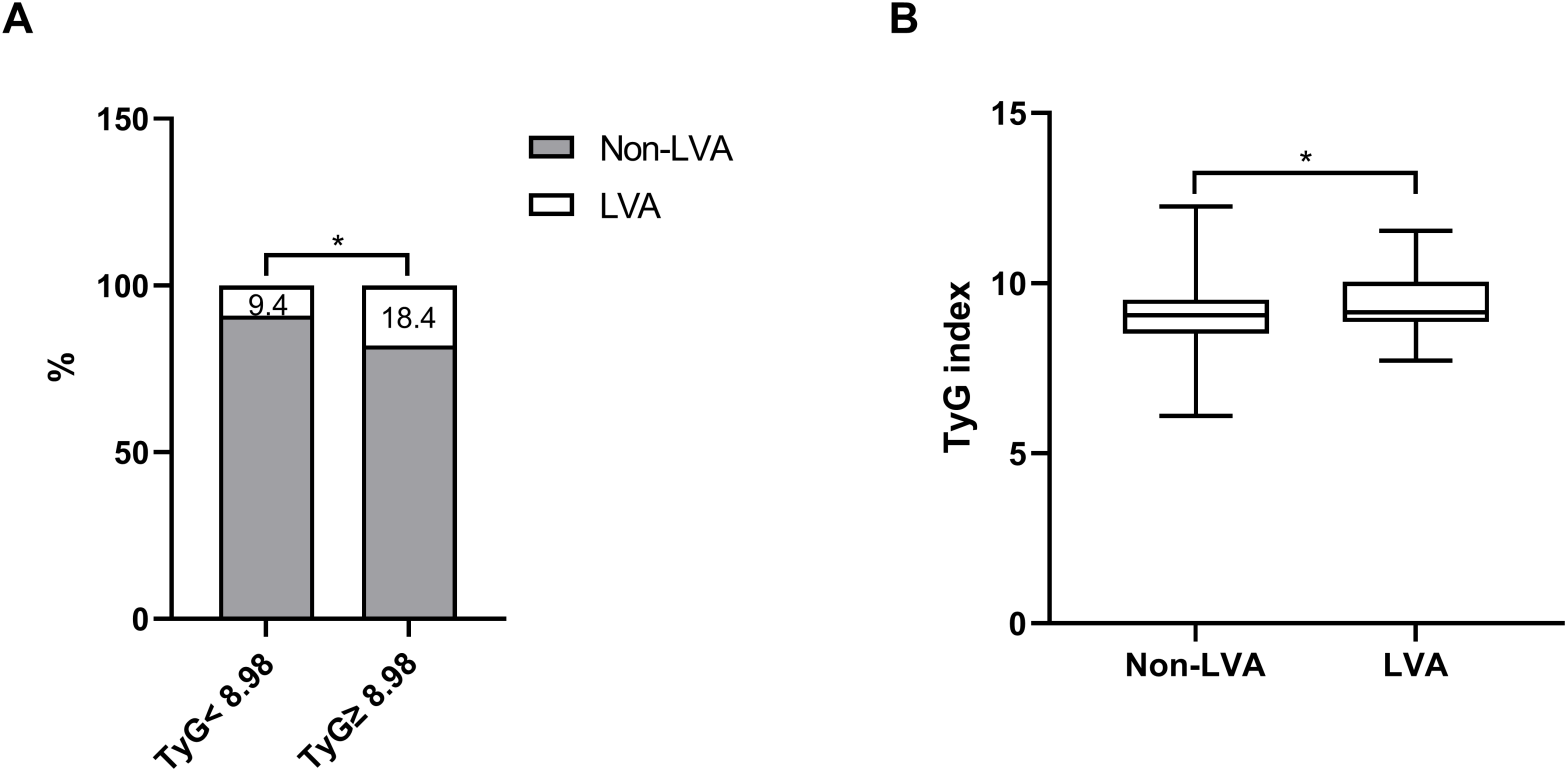
The association between the TyG index and the prevalence of LVA **(A)** and comparison of the TyG index level between the LVA and non-LVA groups **(B)**. TyG, triglyceride-glucose; LVA, left ventricular aneurysm; * P<0.05.

As depicted in **Figure 2A**, the incidence of LVA formation increased with the rise in TyG index (9.4% vs. 18.4%, P< 0.001). Additionally, the LVA group exhibited a significantly higher TyG index than the non-LVA group (9.4 ± 0.8 vs. 9.1 ± 0.8, P < 0.001) (**Figure 2B**).

**Figure 3:**
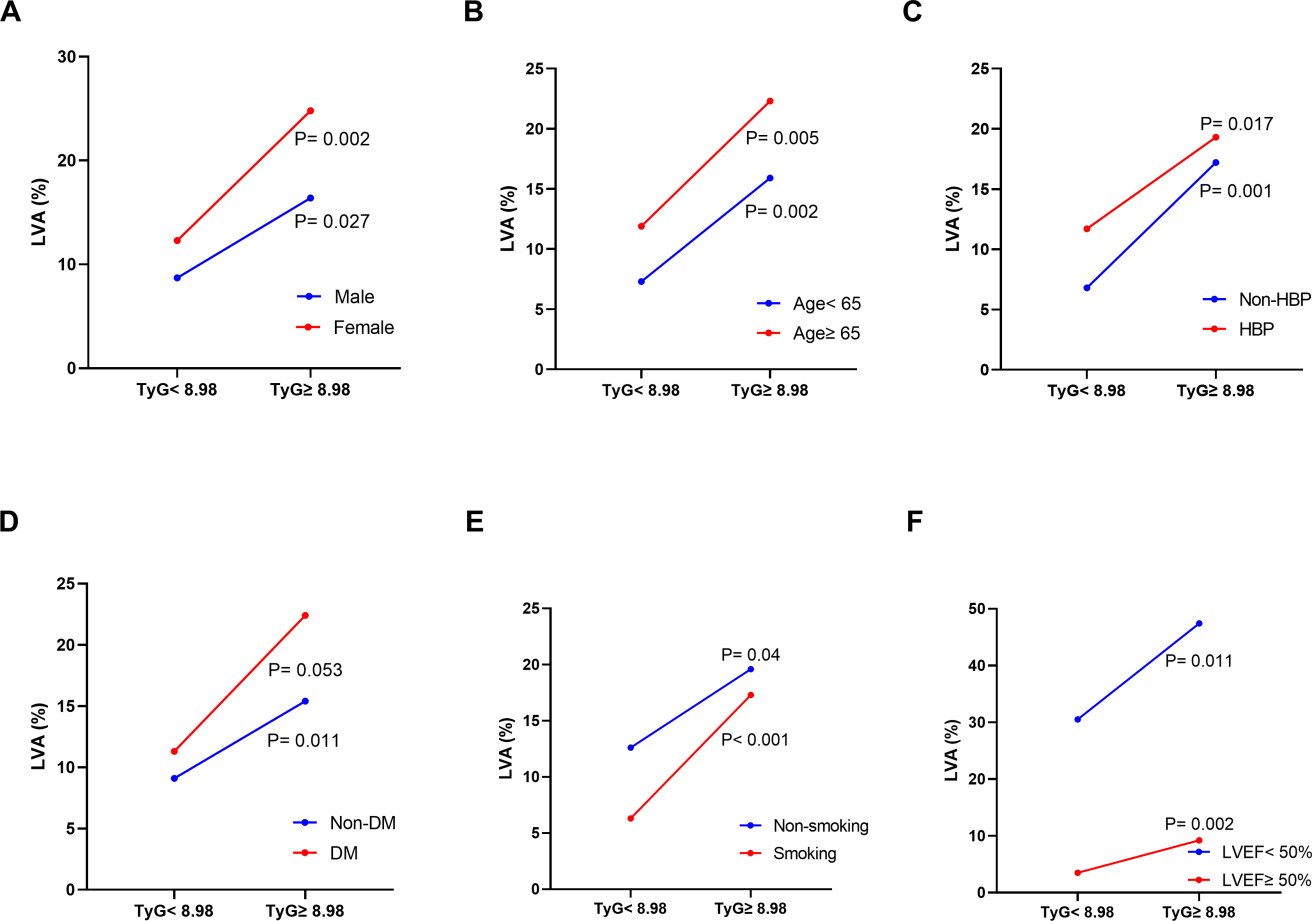
The impact of the TyG index on the prevalence of LVA across subgroups of gender **(A)**, age **(B)**, HBP status **(C)**, DM status **(D)**, current smoking status **(E)**, and LVEF **(F)**. TyG, triglyceride-glucose; LVA, left ventricular aneurysm; HBP, hypertension; DM, diabetes mellitus; LVEF, left ventricular ejection fraction.

**Figure 4:**
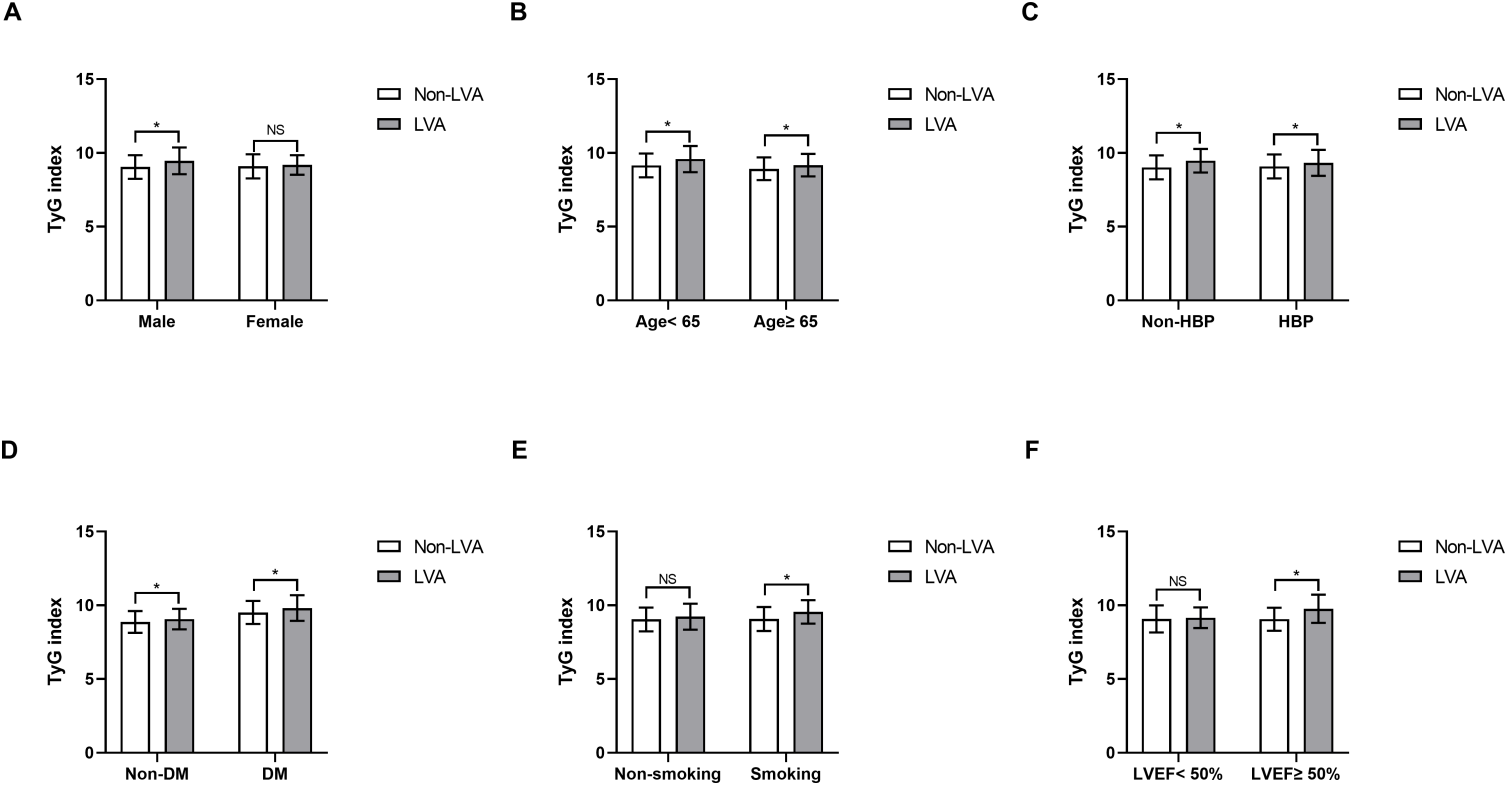
Comparison of the TyG index between non-LVA and LVA groups in the subgroups of gender **(A)**, age **(B)**, HBP status **(C)**, DM status **(D)**, current smoking status **(E)**, and LVEF **(F)**. TyG, triglyceride-glucose; LVA, left ventricular aneurysm; HBP, hypertension; DM, diabetes mellitus; LVEF, left ventricular ejection fraction.

Furthermore, subgroup analysis was performed to assess the association between the TyG index and the incidence of LVA. The prevalence of LVA increased with the rise in TyG index in both males (8.7% vs 16.4%, P= 0.027) and females (12.3% vs 24.8%, P= 0.002), age < 65 years (7.3% vs 15.9%, P= 0.002) and ≥ 65 years (11.9% vs 22.3%, P= 0.005), non-hypertension (6.8% vs 17.2%, P= 0.001) and hypertension (11.7% vs 19.3%, P= 0.017), non-diabetes (9.1% vs 15.4%, P= 0.011), non-smoking (12.6% vs 19.6%, P= 0.04) and smoking (6.3% vs 17.3%, P< 0.001), LVEF < 50% (30.5% vs 47.4%, P= 0.011) and LVEF ≥ 50% (3.5% vs 9.2%, P= 0.002) (**Figures 3A–F**). Simultaneously, the TyG index between the non-LVA and LVA groups in these subgroups was also compared. The results revealed that the TyG index was significantly higher in the LVA group compared to the non-LVA group in all subgroups except for female, patients with non-smoking and LVEF < 50% (**Figures 4A–F**).

### Predictors of LVA formation

Logistic regression analysis was used to evaluate the predictive value of variables for the risk for LVA formation in patients with acute STEMI patients who underwent PCI. Twenty variables were found to be associated with LVA formation in univariate logistic regression analysis (**Table 3**). These variables included age, sex, smoking, LVEF, DBP, heart rate, hemoglobin, AST, HbA1c, TC, TG, LDL-C, LDH, Peak cTnI, FBG, β-blockers use, thiazide or loop diuretic use, gensini score, LAD as the culprit vessel, and TyG index. Subsequently, these 20 variables were included in a multivariate logistic regression analysis, which revealed that only age (OR= 1.04, 95% CI= 1.02-1.07, P= 0.002), LVEF (OR= 0.82, 95% CI= 0.79-0.85, P< 0.001), LDL-C (OR= 2.31, 95% CI= 1.24-4.31, P= 0.008), gensini score (OR= 1.01, 95% CI= 1.01-1.02, P= 0.038), LAD as the culprit vessel (OR= 4.41, 95% CI= 2.42-8.04, P< 0.001), and TyG index (OR= 2.46, 95% CI= 1.64-3.68, P< 0.001) remained significantly associated with the risk for LVA formation.

**Table 3:** Effects of variables on center ventricular aneurysm formation after ST-segment elevation myocardial infarction.

Demographics	Univariate logistic regression analysis			Multivariate logistic regression analysis		
	OR	95% CI	P-value	OR	95% CI	P-value
Age, years	1.04	1.03-1.06	<0.001	1.04	1.02-1.07	0.002
Male, n (%)	1.77	1.20-2.63	0.004			
Hypertension, n (%)	1.36	0.95-1.96	0.095			
Diabetes, n (%)	1.29	0.89-1.88	0.178			
Smoking, n (%)	0.63	0.44-0.90	0.011			
LVEF, %	0.82	0.80-0.85	<0.001	0.82	0.79-0.85	<0.001
SBP, mmHg	1.00	0.99-1.01	0.806			
DBP, mmHg	1.02	1.01-1.03	0.005			
Heart rate, beats/min	1.03	1.02-1.04	<0.001			
White blood cell count, 10^9^/L	1.03	0.98-1.08	0.256			
Red blood cell count, 10^9^/L	0.82	0.62-1.07	0.143			
Neutrophil count, 10^9^/L	1.04	0.99-1.10	0.116			
Platelet count, 10^9^/L	1.00	1.00-1.00	0.222			
Hemoglobin, g/L	0.99	0.98-1.00	0.005			
ALT, U/L	1.00	1.00-1.01	0.721			
AST, U/L	1.00	1.00-1.00	0.028			
HbA1c, %	1.17	1.07-1.29	<0.001			
Cr, umol/L	1.00	0.99-1.00	0.227			
TC, mmol/L	1.15	1.00-1.31	0.044			
TG, mmol/L	0.73	0.61-0.88	<0.001			
HDL-C, mmol/L	1.17	0.61-2.24	0.632			
LDL-C, mmol/L	1.21	1.06-1.39	0.006	2.31	1.24-4.31	0.008
LDH, U/L	1.00	1.00-1.00	<0.001			
Peak cTnI, ng/mL	1.02	1.01-1.02	<0.001			
FBG, mmol/L	1.06	1.02-1.10	0.002			
TyG index	2.17	1.47-3.19	<0.001	2.46	1.64-3.68	<0.001
Statin, n (%)	0.22	0.05-1.00	0.05			
β-blockers, n (%)	0.68	0.46-0.98	0.041			
ACE inhibitors or ARB, n (%)	0.86	0.61-1.23	0.42			
Aldosterone receptor blockers, n (%)	1.34	0.93-1.93	0.115			
Thiazide or loop diuretic, n (%)	1.44	1.00-2.05	0.048			
Gensini Score	1.01	1.01-1.02	<0.001	1.01	1.01-1.02	0.038
Culprit vessel-LAD, n (%)	4.45	2.92-6.77	<0.001	4.41	2.42-8.04	<0.001

LVEF, center ventricular ejection fraction; SBP, Systolic pressure; DBP, Diastolic pressure; ALT, Alanine aminotransferase; AST, Aspartate aminotransferase; HbA1c, glycated hemoglobin; Cr, Creatinine; TC, total cholesterol; TG, triglyceride; HDL-C, high-density lipoprotein cholesterol; LDL-C, low-density lipoprotein cholesterol; LDH, Lactate dehydrogenase; FBG, fasting blood glucose; TyG, triglyceride-glucose; ACE, Angiotensin converting enzyme; ARB, angiotensin receptor blocker; LAD, center anterior descending artery.

Additionally, the study population was randomly divided into a training set (495 patients) and a validation set (496 patients). Multivariate logistic regression analysis demonstrated that the TyG index was significantly and independently associated with the risk for LVA formation in both the training (OR= 2.33, 95% CI= 1.35-4.02, P= 0.002) and validation (OR= 2.68, 95% CI= 1.46-4.92, P= 0.002) set (**Tables 4**, **5**).

**Table 4:** Effects of variables on center ventricular aneurysm formation after ST-segment elevation myocardial infarction in the training set.

Demographics	Univariate logistic regression analysis			Multivariate logistic regression analysis		
	OR	95% CI	P-value	OR	95% CI	P-value
Age, years	1.05	1.03-1.07	<0.001	1.05	1.02-1.09	0.003
Male, n (%)	1.46	0.83-2.57	0.185			
Hypertension, n (%)	1.88	1.13-3.13	0.016			
Diabetes, n (%)	1.30	0.79-2.14	0.3			
Smoking, n (%)	0.64	0.40-1.04	0.074			
LVEF, %	0.82	0.79-0.86	<0.001	0.84	0.80-0.88	<0.001
SBP, mmHg	1.01	1.00-1.02	0.198			
DBP, mmHg	1.02	1.01-1.04	0.006	1.03	1.00-1.06	0.026
Heart rate, beats/min	1.02	1.01-1.04	0.001			
White blood cell count, 109/L	1.03	0.97-1.10	0.369			
Red blood cell count, 109/L	0.82	0.58-1.17	0.282			
Neutrophil count, 109/L	1.05	0.98-1.12	0.161			
Platelet count, 109/L	1.00	1.00-1.00	0.965			
Hemoglobin, g/L	0.99	0.98-1.00	0.014			
ALT, U/L	1.00	0.99-1.00	0.251			
AST, U/L	1.00	1.00-1.00	0.348			
HbA1c, %	1.14	1.01-1.29	0.03			
Cr, umol/L	1.00	1.00-1.01	0.31			
TC, mmol/L	1.13	0.93-1.36	0.22			
TG, mmol/L	0.60	0.44-0.80	0.001			
HDL-C, mmol/L	1.35	0.56-3.23	0.503			
LDL-C, mmol/L	1.26	1.04-1.53	0.021			
LDH, U/L	1.00	1.00-1.00	0.06			
Peak cTnI, ng/mL	1.02	1.01-1.03	0.001			
FBG, mmol/L	1.02	0.96-1.09	0.446			
TyG index	2.03	1.21-3.40	0.007	2.33	1.35-4.02	0.002
Statin, n (%)	0.58	0.06-5.60	0.634			
β-blockers, n (%)	0.66	0.40-1.09	0.104			
ACE inhibitors or ARB, n (%)	0.81	0.50-1.31	0.388			
Aldosterone receptor blockers, n (%)	1.28	0.77-2.11	0.342			
Thiazide or loop diuretic, n (%)	1.58	0.98-2.56	0.062			
Gensini Score	1.01	1.01-1.02	<0.001			
Culprit vessel-LAD, n (%)	3.84	2.23-6.60	<0.001	3.41	1.58-7.39	0.002

LVEF, center ventricular ejection fraction; SBP, Systolic pressure; DBP, Diastolic pressure; ALT, Alanine aminotransferase; AST, Aspartate aminotransferase; HbA1c, glycated hemoglobin; Cr, Creatinine; TC, total cholesterol; TG, triglyceride; HDL-C, high-density lipoprotein cholesterol; LDL-C, low-density lipoprotein cholesterol; LDH, Lactate dehydrogenase; FBG, fasting blood glucose; TyG, triglyceride-glucose; ACE, Angiotensin converting enzyme; ARB, angiotensin receptor blocker; LAD, center anterior descending artery.

**Table 5:** Effects of variables on center ventricular aneurysm formation after ST-segment elevation myocardial infarction in the validation set.

Demographics	Univariate logistic regression analysis			Multivariate logistic regression analysis		
	OR	95% CI	P-value	OR	95% CI	P-value
Age, years	1.03	1.01-1.06	0.006			
Male, n (%)	2.25	1.29-3.93	0.004			
Hypertension, n (%)	0.94	0.56-1.60	0.83			
Diabetes, n (%)	1.24	0.70-2.20	0.458			
Smoking, n (%)	0.60	0.35-1.03	0.065			
LVEF, %	0.82	0.78-0.86	<0.001	0.81	0.76-0.86	<0.001
SBP, mmHg	0.99	0.98-1.00	0.091			
DBP, mmHg	1.01	0.99-1.03	0.248			
Heart rate, beats/min	1.03	1.01-1.05	0.001			
White blood cell count, 10^9^/L	1.03	0.95-1.11	0.494			
Red blood cell count, 10^9^/L	0.78	0.51-1.19	0.255			
Neutrophil count, 10^9^/L	1.03	0.95-1.12	0.453			
Platelet count, 10^9^/L	1.00	0.99-1.00	0.066			
Hemoglobin, g/L	0.99	0.98-1.00	0.123			
ALT, U/L	1.00	1.00-1.01	0.116			
AST, U/L	1.00	1.00-1.00	0.027			
HbA1c, %	1.21	1.04-1.41	0.012			
Cr, umol/L	1.00	1.00-1.01	0.56			
TC, mmol/L	1.18	0.98-1.42	0.078			
TG, mmol/L	0.87	0.70-1.09	0.216			
HDL-C, mmol/L	1.00	0.38-2.65	0.996			
LDL-C, mmol/L	1.19	0.97-1.45	0.093			
LDH, U/L	1.00	1.00-1.00	0.001			
Peak cTnI, ng/mL	1.02	1.00-1.03	0.007			
FBG, mmol/L	1.10	1.04-1.15	<0.001			
TyG index	2.37	1.32-4.26	0.004	2.68	1.46-4.92	0.002
Statin, n (%)	0.07	0.01-0.79	0.031			
β-blockers, n (%)	0.71	0.40-1.24	0.228			
ACE inhibitors or ARB, n (%)	0.94	0.55-1.59	0.809			
Aldosterone receptor blockers, n (%)	1.46	0.85-2.50	0.168			
Thiazide or loop diuretic, n (%)	1.26	0.74-2.16	0.400			
Gensini Score	1.01	1.01-1.02	0.001			
Culprit vessel-LAD, n (%)	5.61	2.85-11.05	<0.001	8.3	3.07-22.46	<0.001

LVEF, center ventricular ejection fraction; SBP, Systolic pressure; DBP, Diastolic pressure; ALT, Alanine aminotransferase; AST, Aspartate aminotransferase; HbA1c, glycated hemoglobin; Cr, Creatinine; TC, total cholesterol; TG, triglyceride; HDL-C, high-density lipoprotein cholesterol; LDL-C, low-density lipoprotein cholesterol; LDH, Lactate dehydrogenase; FBG, fasting blood glucose; TyG, triglyceride-glucose; ACE, Angiotensin converting enzyme; ARB, angiotensin receptor blocker; LAD, center anterior descending artery.

### Prognostic analysis

**Figure 5:**
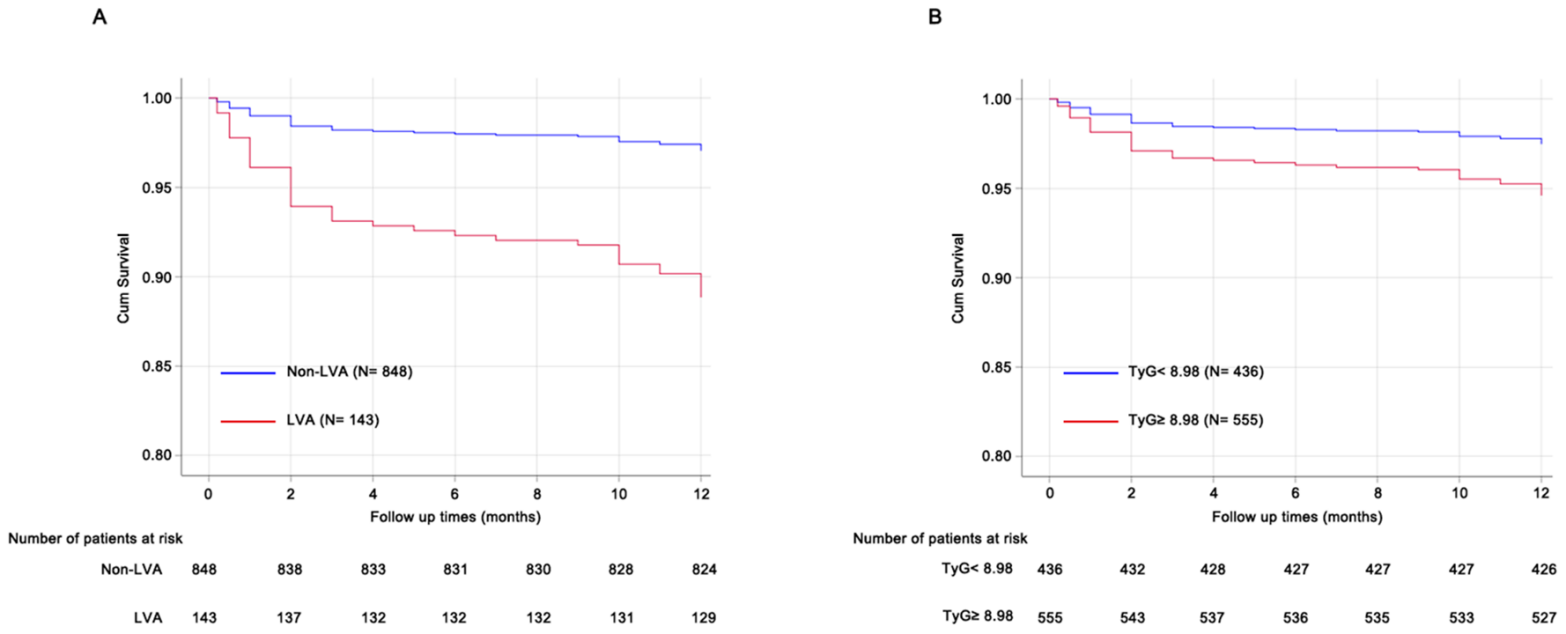
Prognosis analysis using Cox proportional hazard regression models. **(A)** patients with LVA was associated with poorer prognosis compared to those without LVA. **(B)**, patients with TyG≥ 8.98 showed increased cardiac death risk compared to those with TyG< 8.98. TyG, triglyceride-glucose; LVA, left ventricular aneurysm.

During the follow-up period, a total of 41 cardiac death occurred. The number of cardiac death in the non-LVA group, the LVA group, the TyG< 8.98 group, and TyG≥ 8.98 group were 25 (2.9%), 16 (11.2%), 11 (2.5%), and 30 (5.4%), respectively. Cox proportional hazard regression analysis indicated that patients with LVA had an increased cardiac death risk compared to those without LVA (HR= 3.94, 95% CI= 2.11-7.39; P < 0.001) (**Table 6**, **Figure 5A**). Furthermore, patients with TyG≥ 8.98 was associated with worse prognosis compared to those with TyG< 8.98 (HR= 2.17, 95% CI= 1.09-4.33; P= 0.04) (**Table 6**, **Figure 5B**). These statistical significances remained even after adjustments for sex, age, hypertension, diabetes, smoking state, and β‐blocker use (**Table 6**).

**Table 6:** Prognosis analysis using cox proportional hazard regression model.

Model	Non-LVA vs. LVA		TyG< 8.98 vs. TyG≥ 8.98	
	HR (95% CI)	P-value	HR (95% CI)	P-value
Unadjusted	3.94 (2.11-7.39)	<0.001	2.17 (1.09-4.33)	0.04
Adjusted	3.16 (1.67-5.98)	<0.001	2.39 (1.09-5.23)	0.03

Hazard Ratio (HR) and 95% confidence intervals (95% CI) were obtained by cox proportional hazard regression, without and with adjustment for sex, age, hypertension,diabetes, smoking status and β-blocker use. LVA, center ventricular aneurysm.

### Association between TyG index and clinical parameters of LVA

**Figure 6:**
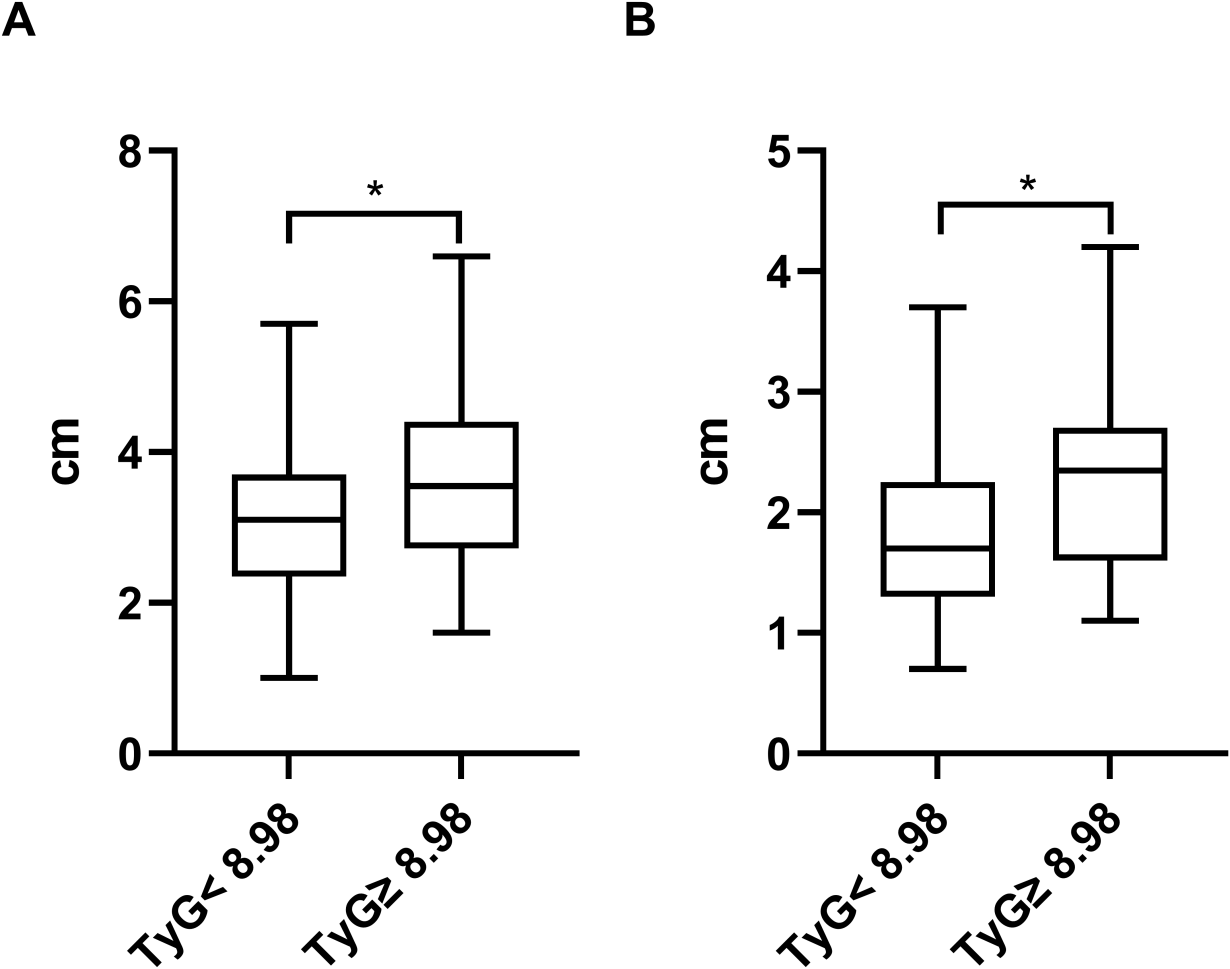
Comparison of the maximal length **(A)** and width **(B)** of LVA between patients grouped by TyG index. TyG, triglyceride-glucose; *P<0.05.

The study aimed to investigate the relationship between the TyG index and the size of LVA by comparing the maximal length and width of LVA in patients with TyG< 8.98 and TyG≥ 8.98. Both maximal length and width were significantly greater in patients with TyG ≥ 8.98 compared to those with TyG < 8.98 (maximal length, 3.04 ± 0.96 vs. 3.55 ± 1.15; maximal width, 1.89 ± 0.70 vs. 2.34 ± 0.86) (**Figures 6A, B**).

### ROC curve analysis

**Figure 7:**
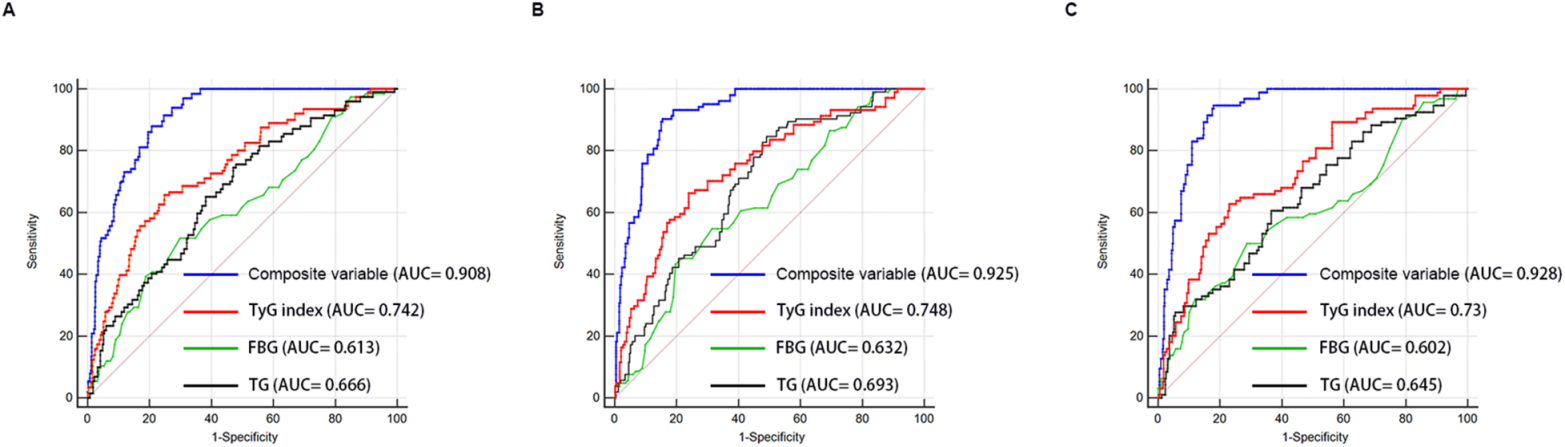
Receiver-operating characteristic curves for prediction of LVA formation in the whole cohort **(A)**, the training set **(B)**, and the validation set **(C)**. AUC, area under the curve; TyG, triglyceride-glucose; FBG, fasting blood glucose; TG, triglyceride.

ROC curve analysis was used to assess and compare the predictive abilities of TG, FBG, TyG index, and the composite variable (TyG index combined with LVEF and LAD as the culprit vessel). As shown in **Figure 7A** and **Table 7**, the average area under the ROC curve (AUC) for TG, FBG, TyG index, and the composite variable were 0.666 (95% CI = 0.641 - 0.690), 0.613 (95% CI = 0.587 - 0.638), 0.742 (95% CI= 0.719 - 0.764), and 0.908 (95% CI = 0.892 - 0.922), respectively. Furthermore, the TyG index demonstrated significantly higher AUCs for predicting the risk for LVA formation compared with TG (P < 0.001) and FBG (P < 0.001). Additionally, the composite variable exhibited the highest predictive value (P < 0.001). To avoid overfitting, cross-validation was conducted by randomly dividing the population into the training and the validation sets. As shown in **Figure 6B, C** and **Table 6**, the TyG index displayed superior discriminative power compared with both TG and FBG in both the training and validation sets (training set: P= 0.041 for TyG index vs. TG; P < 0.001 for TyG index vs. FBG; validation set: P= 0.002 for TyG index vs. TG; P < 0.001 for TyG index vs. FBG). Clearly, the composite variable demonstrated the highest predictive value in both the training and validation sets.

**Table 7:** Analysis of the ROC curve for predictive power of center ventricular aneurysm formation.

Cohort	Variables	AUC	SE	95% CI
Whole cohort	FBG	0.613	0.022	0.587 - 0.638
	TG	0.666	0.02	0.641 - 0.690
	TyG index	0.742	0.019	0.719 - 0.764
	Composite variable	0.908	0.008	0.892 - 0.922
Training set	FBG	0.632	0.028	0.596 - 0.668
	TG	0.693	0.026	0.658 - 0.727
	TyG index	0.748	0.027	0.715 - 0.779
	Composite variable	0.925	0.011	0.903 - 0.943
Validation set	FBG	0.602	0.034	0.565 - 0.638
	TG	0.645	0.031	0.608 - 0.680
	TyG index	0.73	0.028	0.696 - 0.763
	Composite variable	0.928	0.01	0.906 - 0.946

ROC, receiver operating characteristic; FBG, fasting blood glucose; TG, triglyceride; TyG, triglyceride-glucose; AUC, area under curve; SE, standard error; CI, Confidence interval.

Additionally, we evaluated the predictive capabilities of LDH, LDL-C, and the TyG index concerning the risk of LVA formation. As illustrated in **Supplementary Table 1**, the TyG index exhibited superior predictive performance compared to both LDH (P < 0.001) and LDL-C (P < 0.001) in assessing the risk of LVA formation. Furthermore, the TyG index could improve the discriminatory capability of the composite of LVEF and LAD as a culprit vessel for identifying the development of LVA (**Supplementary Table 2**) (P< 0.001).

### Subgroup analyses

**Figure 8:**
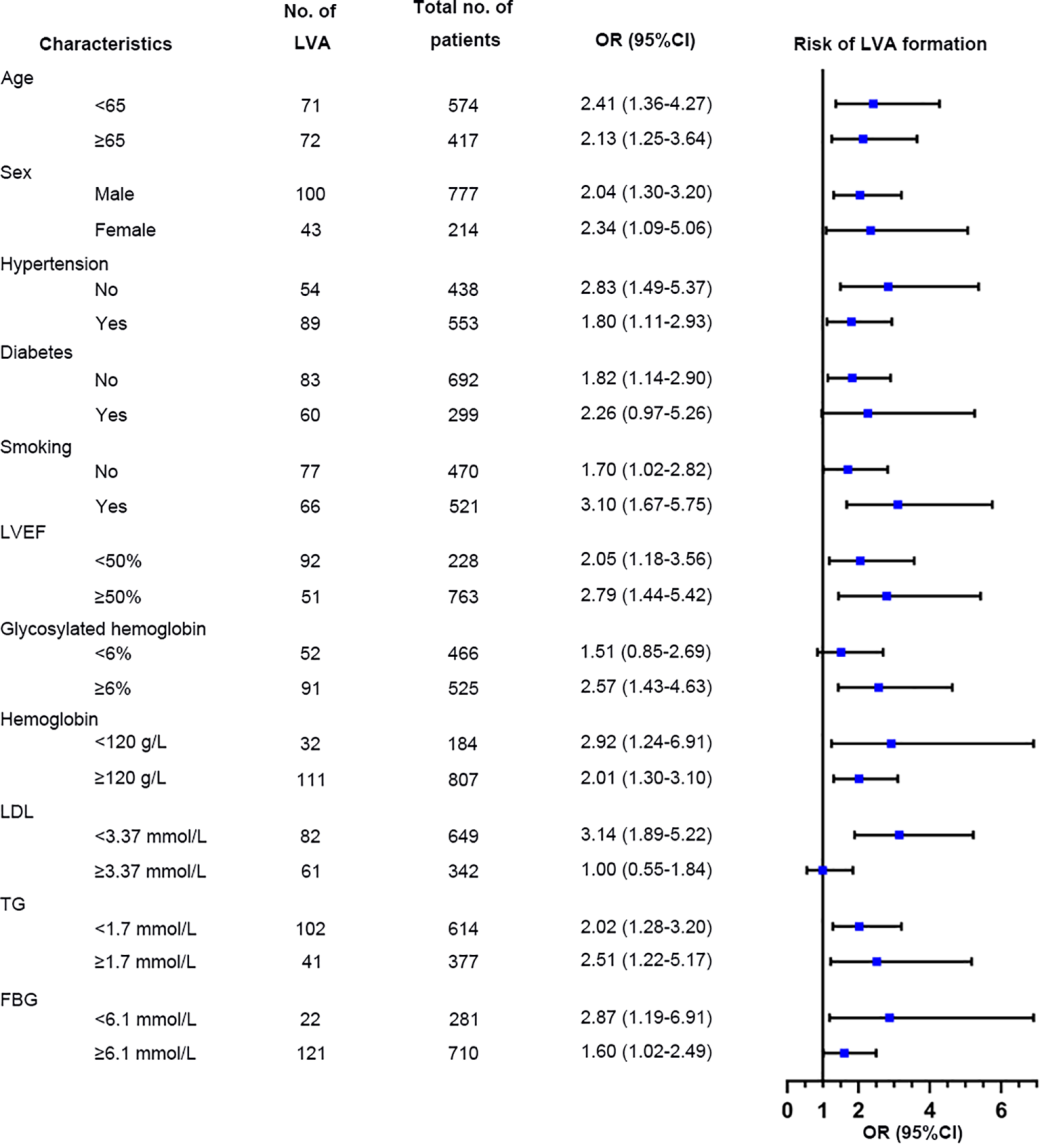
Forest plots for subgroup analysis of the relationship between TyG index and the risk for LVA formation. TyG, triglyceride-glucose; LVA, left ventricular aneurysm; LVEF, left ventricular ejection fraction; LDL-C, low-density lipoprotein cholesterol; TG, triglyceride; FBG, fasting blood glucose; OR, Odds Ratio.

Additional analyses were performed on several subgroups to evaluate the independent predictive value of the TyG index for LVA formation. The independent predictive effect of the TyG index on LVA formation was primarily represented in the subgroups of age< 65 and ≥65 years, male and female, with and without hypertension, without diabetes, with and without smoking, LVEF< 50% and LVEF≥ 50%, HbA1c≥ 6%, hemoglobin< 120g/L and ≥ 120g/L, LDL-C< 3.37mmol/L, TG <1.7mmol/L and ≥1.7 mmol/L, FBG <6.1 mmol/L and ≥6.1 mmol/L (**Figure 8**). The association between the TyG index and LVA formation showed no statistically significance in subgroup analysis of patients with diabetes, HbA1c< 6%, and LDL-C≥3.37mmol/L.

## Discussion

In the present study, we investigated the predictive value of TyG index for LVA formation and prognosis in patients with STEMI. The results indicated that a higher TyG index independently predicted LVA formation. The predictive value remained significant in both the training and validation sets. Besides, higher TyG index was correlated with increased cardiac death risk. Among patients with LVA, those with a TyG index≥ 8.98 exhibited significantly larger maximal length and width compared to those with a TyG index < 8.98. The relationship between the TyG index and LVA formation was generally consistent across subgroups, except for patients with diabetes, HbA1c< 6%, and LDL≥3.37mmol/L. Additionally, the TyG index exhibited greater predictive power for LVA formation than both TG and FBG. However, the composite variable exhibited the best predictive value.

As a hallmark of type 2 diabetes mellitus, insulin resistance (IR) refers to a state of reduced sensitivity and responsiveness to the action of insulin (17). Multiply studies have demonstrated that IR was associated with the progression of cardiovascular diseases and could predict cardiovascular outcomes (18–21). The euglycemic insulin clamp and intravenous glucose tolerance testing are the gold standards for IR (22). However, they are not suitable for clinical practice due to invasiveness and high cost. Currently, the homeostasis model assessment-estimated insulin resistance (HOMA-IR) index is widely used for assessing β-cell function and IR. Nevertheless, its complexity and time-consuming nature limit its application in practical clinical settings and large-scale studies (23).

The TyG index has emerged as a reliable marker of IR and has been shown to be superior to HOMA-IR in assessing metabolic syndrome (24, 25). TyG is a simple, convenient and low-cost clinical index that can easily be obtained for large-scale study. Numerous studies have demonstrated the association between TyG index and the cardiovascular diseases (8, 11, 26, 27). In a prospective study including 1574 patients with acute coronary syndrome (ACS), Zhu et al. found that an elevated TyG index was independently and positively associated with in-stent restenosis after drug-eluting stent (28). A study by Wang et al. investigated 2531 consecutive patients with diabetes who underwent coronary angiography for ACS and demonstrated TyG index to be an independent predictor for the major adverse cardiovascular events (10). Furthermore, a higher TyG index was found associated with the presence of a higher coronary anatomical complexity in ACS patients (29). However, no study has investigated the association among the TyG index, LVA formation, and the prognosis of patients with STEMI. In the present study, we demonstrated for the first time that TyG index≥ 8.98 was significantly associated with increased LVA formation and cardiac death risk compared to TyG index < 8.98. Importantly, the risk for LVA formation in patients with TyG index≥ 8.98 exceeded 2-fold compared to those with TyG index< 8.98.

As a common and severe complication of AMI, LVA is characterized by the outward expansion of the infarcted myocardium during systole and diastole (30). Recently, the incidence of LVA after acute STEMI has decreased from 10-30% to 8-15% due to advancements in the treatment for AMI (16). In our study, the incidence of LVA in acute STEMI patients who underwent primary PCI was approximately 14.4%, which aligns with previous reports (3, 5). In fact, numerous studies have aimed to identify predictors for LVA after AMI. Feng et al. found that single-vessel disease, decreased GFR and abnormal ferritin could independently predict the LVA formation (31). In a retrospective study involving 1823 STEMI patients, Zhang et al. found that female sex, peak NT-pro BNP, the time between the onset of pain and balloon time, presence of QS-waves on initial electrocardiogram were the independent predictors of early-onset LVA (32). In a prospective cohort study including 1519 patients with STEMI, Savas et al. found that plasma N- Terminal pro B type natriuretic peptide level at admission, among other variables, provided valuable predictive information regarding the development of LVA (16). Additionally, a cohort study by Zhang et al. identified the blood urea nitrogen-to-albumin ratio as an independent predictor for LVA formation in STEMI patients with primary PCI (33). However, disagreement persists concerning these risk factors among different cohort studies. Moreover, these studies primarily focused on single variables, most of which had only modest or small effects on LVA prediction. In the present study, we introduced the TyG index, for the first time, to predict LVA formation in patients with STEMI. The OR for LVA formation in patients with TyG index≥ 8.98 reached up to 2.46. Patients with a TyG index ≥ 8.98 exhibited a significant increase in maximal length and width compared to those with a TyG index < 8.98, which demonstrated the critical predictive role of TyG index in myocardial injury. In addition, LVEF was also identified as the independent predictor for LVA formation, which is consistent with the previous study (5). Furthermore, the TyG index proved to be a more potent predictor for LVA formation than both TG and FBG, with the composite variable yielding the highest predictive value.

The precise mechanisms underlying the relationship between the TyG index and LVA formation remain incompletely elucidated. Firstly, the TyG index exhibits positive correlations with TC, LDL-C, and C-reactive protein, while showing a negative correlation with HDL-C. This suggests that the presence of cardiometabolic risk factors may partially account for this association. Secondly, the TyG index serves as a reliable indicator of insulin resistance (IR). Prior research has indicated that IR can lead to inflammation, oxidative stress, and cardiomyocyte apoptosis (34, 35), potentially elevating the risk of LVA. Thirdly, an elevated TyG index has been associated with increased arterial stiffness and coronary artery calcification (13, 36), which may represent another significant mechanism.

The association between the TyG index and LVA formation was not observed in patients with diabetes, those with HbA1c HbA1c< 6%, or those with LDL-C≥ 3.37mmol/L. The limited sample size of diabetic patients (N=299) may account for the lack of significant association between the TyG index and LVA. Patients with HbA1c < 6% demonstrated a lower incidence of LVA compared to those with HbA1c ≥ 6% (11.2% vs. 17.3%, P<0.001), potentially diminishing the impact of the TyG index on LVA risk in this subgroup. Additionally, the number of patients with LDL-C≥ 3.37mmol/L was significantly smaller than those with LDL-C< 3.37mmol/L. Furthermore, patients with LDL-C≥ 3.37mmol/L were younger than those with lower LDL-C levels (59.7 ± 13.3 vs. 61.9 ± 12.5, P=0.01) in our study. These factors collectively attenuate the relationship between the TyG index and LVA.

The primary strength of the present study is its novelty as the first investigation addressing the relationship between TyG index and the risk for LVA formation in patients with acute STEMI who underwent primary PCI. However, several limitations should be noted. First, our study failed to compare the predictive values of HOMA-IR and the TyG index due to the lack of routine insulin level measurements in these patients. Second, despite the large sample size in this study, our focus was solely on baseline serum TG and FBG levels, neglecting the dynamic changes in the TyG index, which could have provided valuable insight into the underlying mechanism. Third, the nature of observational study prevented the establishment of a causal association between the TyG index and LVA formation. Additionally, unmeasured or residual confounding effects may have impacted our findings. Finally, while our results indicated a significant association between the TyG index and the risk for LVA formation, its practical clinical application value requires confirmation in future prospective studies.

## Conclusions

In conclusion, our study demonstrated that the TyG index was significantly associated with increased risk of LVA formation and cardiac death in patients with acute STEMI who underwent primary PCI. Additionally, the TyG index alone demonstrated excellent discriminatory ability for LVA formation, and the composite variable including the TyG index, LVEF, and LAD as the culprit vessel significantly improved the discriminatory power. Our findings suggest the potential use of the TyG index in clinical practice as a preferable predictor for LVA formation in patients with acute STEMI who underwent primary PCI.

## Data Availability

The raw data supporting the conclusions of this article will be made available by the authors, without undue reservation.
